# Evaluation of Resin-Based Material Containing Copaiba Oleoresin (*Copaifera Reticulata* Ducke): Biological Effects on the Human Dental Pulp Stem Cells

**DOI:** 10.3390/biom10070972

**Published:** 2020-06-28

**Authors:** Roberta Souza D’Almeida Couto, Maria Fernanda Setubal Destro Rodrigues, Leila Soares Ferreira, Ivana Márcia Alves Diniz, Fernando de Sá Silva, Talita Christine Camilo Lopez, Rafael Rodrigues Lima, Márcia Martins Marques

**Affiliations:** 1 Department of Restorative Dentistry, School of Dentistry, University of Sao Paulo, São Paulo, SP 05508-060, Brazil; leilasfer@yahoo.com.br (L.S.F.); marcia.marques@ibirapuera.edu.br (M.M.M.); 2 School of Dentistry, Federal University of Pará, Belém, PA 66075-110, Brazil; 3 Postgraduation Program in Biophotonics Applied to Health Sciences, Nove de Julho University, São Paulo, SP 02112-000, Brazil; fernandarodrigues@usp.br (M.F.S.D.R.); tal_lopez@hotmail.com (T.C.C.L.); 4 Department of Restorative Dentistry, School of Dentistry, Federal University of Minas Gerais, Belo Horizionte, MG 31270-901, Brazil; ivanadiniz@ymail.com; 5 Departamento de Ciências da Vida, Federal University of Juiz de Fora, Juiz de Fora, MG 36036-900, Brazil; silvafs@gmail.com; 6 Institute of Biological Sciences, Federal University of Pará, Belém, PA 66075-110, Brazil; rafalima@ufpa.br; 7 Post graduation course in Dentistry, Ibirapuera University, São Paulo, SP 04661-100, Brazil

**Keywords:** stem cells, dental pulp, MTA, pulp capping, pulp repair

## Abstract

The purpose of this study was to analyze in vitro the biological effects on human dental pulp stem cells triggered in response to substances leached or dissolved from two experimental cements for dental pulp capping. The experimental materials, based on extracts from *Copaifera reticulata* Ducke (COP), were compared to calcium hydroxide [Ca(OH)_2_] and mineral trioxide aggregate (MTA), materials commonly used for direct dental pulp capping in restorative dentistry. For this, human dental pulp stem cells were exposed to COP associated or not with Ca(OH)_2_ or MTA. Cell cytocompatibility, migration, and differentiation (mineralized nodule formation (Alizarin red assay) and gene expression (RT-qPCR) of *OCN*, *DSPP*, and *HSP-27* (genes regulated in biomineralization events)) were evaluated. The results showed that the association of COP reduced the cytotoxicity of Ca(OH)_2_. Upregulations of the *OCN*, *DSPP*, and *HSP-27* genes were observed in response to the association of COP to MTA, and the *DSPP* and *HSP-27* genes were upregulated in the Ca(OH)_2_ + COP group. In up to 24 h, cell migration was significantly enhanced in the MTA + COP and Ca(OH)_2_ + COP groups. In conclusion, the combination of COP with the currently used materials for dental pulp capping [Ca(OH_)2_ and MTA] improved the cell activities related to pulp repair (i.e., cytocompatibility, differentiation, mineralization, and migration) including a protective effect against the cytotoxicity of Ca(OH)_2_.

## 1. Introduction

Restoring lost dental hard tissue without compromising the vitality of the tooth has been a significant challenge for the restorative dentistry [1]. In this regard, direct pulp capping is the oldest procedure for the treatment of deep caries lesions or even fractures that compromise the dental pulp integrity [2,3]. The materials used for direct pulp capping aiming at pulp repair [4] are mainly calcium hydroxide-based [5,6,7,8,9,10,11]. However, calcium hydroxide-based materials only induce the formation of low-quality mineralized tissue barriers [6,7]. Mineral trioxide aggregate (MTA) is another material used for this purpose; however, it also has limitations in its use with regard to the darkening of teeth and its high cost, among others.

A systematic review of the current trends and future perspectives of dental pulp capping materials have shown that advances in bioactive materials, especially those developed from MTA, are promising to improve biomaterials for application in the treatment of vital pulp [8]. Additionally, an overview on the MTA and other bioactive endodontic cements called attention to the high number of new bioactive endodontic cement that have been introduced to market. However, the number of studies on these materials compared to investigations on MTA is limited [9]. This indicates the need for studies testing bioactive materials for the treatment of exposed vital dental pulp. Moreover, copaiba has not been mentioned in any of the searched studies in those reviews, showing the novelty of the present study.

In contemporary dentistry, it is clear that an ideal dental capping material has yet to be developed. Such material should limit the inflammatory response of the exposed pulp, and accelerate pulp repair, leading to the deposition of mineralized dentin of physiological quality [12]. Copaiba (COP) has been used for many years due to its anti-inflammatory, antiseptic, and analgesic properties [13,14]. Copaiba oleoresin is obtained by tapping the trunk of the genus *Copaifera* tree, and the most widely used species used by traditional communities in the Brazilian Amazon region is *C. reticulata* Ducke. This oleoresin has resinous acids and volatile compounds in its chemical composition as several sesquiterpenes including β-caryophyllene (bactericidal and anti-inflammatory), β-bisabolene (anti-inflammatory), and α-humulene (anti-inflammatory) [13,14]. In recent studies, the anti-inflammatory and scarring induction effects promoted by copaiba in oral mucosa injuries has been confirmed [15,16,17].

It is also known that the non-contaminated exposed dental pulp tissue responds with the formation of a dentin bridge. This dentin is formed by mesenchymal undifferentiated cells that proliferate, migrate toward the exposed area, and differentiate into odontoblast-like cells able to secrete dentin extracellular matrix [18]. The biological and physical characteristics of the COP oleoresin raised the hypothesis that the association between COP and materials commonly used for pulp capping would result in more bioactive materials that in turn would induce improvement in the regenerative potential of mesenchymal undifferentiated cells of the dental pulp. 

Thus, the purpose of the present study was to analyze the in vitro biological responses of human dental pulp stem cells submitted to COP associated or not with Ca(OH)_2_ or MTA, materials that are commonly used in restorative dentistry.

## 2. Materials and Methods

### 2.1. Cell Culture

**Figure 1:**
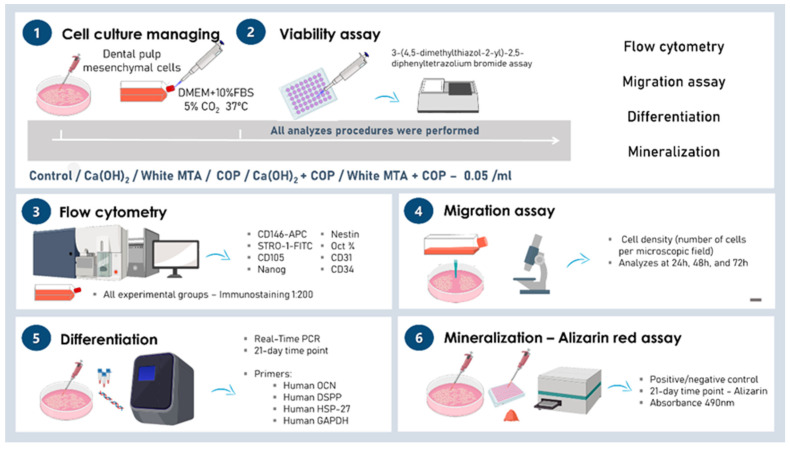
Methodological figure.

Human mesenchymal stem cells (hMSCs) derived from dental pulp of an exfoliated deciduous tooth were used in this study. This study was approved by the Human Research Ethics Committee of the School of Dentistry, University of São Paulo under Protocol #106/11CAAE 0116.0.017.000-11. The cells were cultured in a clonogenic culture medium composed of DMEM/Ham’s F-12 (1:1, LGC Biotecnologia, Cotia, SP, Brazil), supplemented with 15% fetal bovine serum (FBS, Hyclone, Thermo Scientific, South Logan, UT, USA), 100 U/mL penicillin (Invitrogen/Gibco, Grand Island, NY, USA), 100 μg/mL streptomycin (Invitrogen/Gibco), 2 mM L-glutamine (Invitrogen/Gibco), and 2 mM nonessential amino acids (Invitrogen/Gibco). The cells were maintained in a 5% CO_2_ incubator at 37 °C. All experiments are described in Figure 1.

### 2.2. Flow Cytometry

Cells were characterized by flow cytometry using CD146-APC and STRO-1-FITC (Biolegend, San Diego, CA, USA), CD105 (Santa Cruz Biotechnology, Santa Cruz, CA, USA), Nanog (Santa Cruz Biotechnology), Nestin (Santa Cruz Biotechnology), Oct3/4 (Santa Cruz Biotechnology), CD31 (Santa Cruz Biotechnology), and CD34 (Santa Cruz Biotechnology) antibodies. Cells were fixed in 4% paraformaldehyde for 1 h, incubated in bovine serum albumin (BSA) for 30 min, and incubated with the target antibodies (1:200 dilution) at 4 °C for 30 min. A total of 50,000 events were gathered (FACs Calibur, BD, Franklin Lakes, NJ, USA) and sorted using FlowJo software (Tree Star, Aschland, OR, USA).

### 2.3. Biomaterials

The *Copaifera reticulata* Ducke (COP) *in natura* was kindly provided by the Laboratory of Functional and Structural Biology, Federal University of Pará, Belém-Pa, Brazil. COP was collected by exudation from the trunk of a tree C. reticulata Ducke located in the municipality of Belterra, Pará, Brazil (Latitude: 02°38′11” S, Longitude: 54°56′14” W) that was approximately 30 years old. After collection, the oleoresin was stored in the absence of light, oxygen, and heat in order to keep its volatile compounds stabilized. A sample of the plant was conserved in the herbarium of the Brazilian Agricultural Research Corporation (Embrapa, Belém, PA, Brazil), under registration number 183939, following international guidelines suggested by the World Health Organization (WHO). This oleoresin has been used in several studies published by our group in recent years [15,16,17,18]. The chromatographic characterization was previously published, and periodically validated for each study, with no significant differences between the percentage of its components over the years. The phytochemical characterization is described in Guimarães-Santos et al. (2012) [19].

COP was used *in natura* in association with calcium hydroxide [Ca(OH)_2_] or in association with mineral trioxide aggregate (MTA). To obtain the combined materials, COP was mixed to powder of Ca(OH)_2_ or MTA to obtain pastes according to the patented formulations (INPI BR 10 2013 005551-4). All information is summarized in Table 1. To obtain the pastes, the Ca(OH)_2_ and white MTA were prepared according to the manufacturer’s recommendations, where the powder was mixed with distilled water. 

### 2.4. Conditioned Media and Experimental Groups

To mimic in vivo conditions where the dental pulp stem cells are in contact with substances leached or dissolved from the pulp capping materials into dental fluids, the biomaterials were placed indirectly in contact with the stem cell cultures by applying conditioned culture media. Conditioned media were obtained by incubating 0.05 g of each substance per mL of fresh clonogenic medium for 1 h at 37 °C. The conditioned medium was then filtered through a 0.22 µm filter and immediately applied to the cell cultures, according to the experimental groups: control group (CG), cells grown in fresh medium; COP, cells grown in medium conditioned with copaiba oil-resin; Ca(OH)_2_, cells grown in medium conditioned with calcium hydroxide; Ca(OH)_2_ + COP, cells grown in medium conditioned with Calcium hydroxide associated with copaiba oleoresin; MTA: cells grown in medium conditioned with white MTA cement; and MTA + COP, cells grown in medium conditioned with white MTA cement associated with copaiba oleoresin.

### 2.5. Cell Viability

Cells were plated (2 × 10^3^ cells per well) in 48-well culture plates. The cell viability of all groups was measured at 24 h, 48 h, and 72 h after the application of the conditioned media. To infer the cell viability and to plot the cell growth curves, the mitochondrial activity was analyzed using the 3-(4,5-dimethylthiazol-2-yl)-2,5-diphenyltetrazolium bromide assay (Molecular Probes, Life Technologies Corporation, Grand Island, NY, USA), following the manufacturer’s instructions. The optical density was determined in a microplate reader (Synergy HT, Bio-TeK^®^ Instrument Inc., Winooski, VE, USA) using a 562 nm filter.

### 2.6. Migration

Cells were plated (5 × 10^5^ cells per well) in 6-well culture plates. After confluence, each monolayer was submitted to the “scratch” assay using a p200 pipette tip. The wound closure process was recorded 0, 12, 24, and 48 h later. The cell density (number of cells per microscopic field) in the wound area was used to compare migration amongst the groups at the same experimental times.

### 2.7. Differentiation: Quantitative Real-Time Polymerase Chain Reaction (RT-qPCR)

Cells were plated in 100 mm diameter dishes (3 × 10^5^ cells per dish). Twenty-four hours after seeding, the media were replaced with the experimental media. At the 21-day time point, the total mRNA was extracted from homogenized cells with TRIzol (Invitrogen) to analyze the levels of gene expression. The RT-qPCR reactions were performed using an Applied Biosystems 7500 real-time PCR system with SYBR Green I Dye (Applied Biosystems, Foster City, CA, USA), according to the manufacturer’s instructions. The following primers were used: human OCN (sense 5′-cgctaacctgtatcaatggctgg-3′ and antisense 5′-ctcctgaaagccgatgtggtca-3′; size = 123 bp); human DSPP (sense 5′- caaccatagagaaagcaaacgc-3′ and anti-sense 5′-tttctgttgccactgctgggac-3′; size = 120 bp); human *HSP-27* (sense 5′-acggtcaagaccaaggat-3′ and anti-sense 5′-agcgtgtatttccgcgtg-3′; size = 104 bp), and human GAPDH (sense 5′- gcatcctgggctacactga-3′ and anti-sense 5′- ccaccaccctgttgctgta-3′; size = 162 bp).

### 2.8. Mineralization: Mineralized Matrix Formation (Alizarin Red Assay)

For functional cell differentiation assay, cells were cultured in inductive mineralizing medium composed of DMEM/Ham’s F-12, supplemented with 15% SFB (Hyclone), 50 mg/mL penicillin-streptomycin-glutamine (PSG, Invitrogen), 50 µg ascorbic acid (Sigma, St. Louis, MO, USA), 1 mM β- glycerophosphate (Sigma), and 10 nM dexamethasone (Sigma). Two experimental groups were included for this analysis: positive control (cells grown in fresh mineralizing culture medium) and negative control (cells grown in fresh clonogenic culture medium). Cells were plated (5 × 10^3^ cells per well) in 48-well plates. Twenty-four hours after seeding, the media were replaced with the experimental media. At the 21-day time point, other cultures were subjected to the Alizarin red S assay to detect possible mineralized nodules. Absorbance was then measured at the wavelength of 490 nm.

### 2.9. Statistical Analysis

Quantitative data on cytocompatibility, differentiation, mineralization, and migration in the experimental groups were compared using analysis of variance (ANOVA), complemented with Tukey’s test (*p* ≤ 0.05).

## 3. Results

### 3.1. The Dental Pulp Cells Presented a Mesenchymal Stem Cell Immunophenotype

**Figure 2:**
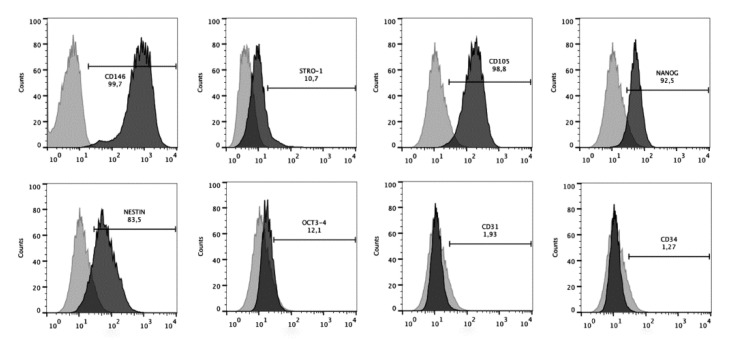
hMSCs characterization immunoprofile of dental pulp stem cells. Positive expressions of CD146, STRO-1, CD105, Nanog, Nestin, and Oct3/4, and negative or minimum expression of CD31 and CD34.

The stem cell nature of dental pulp cells was confirmed by their immunoprofile observed in flow cytometry (Figure 2). Cells showed high positive expression of surface markers of the mesenchymal stem cells CD146 (99.7%), CD105 (98.8%), Nanog (92.5%), and Nestin (83.5%), and low expression of Stro-1 (10.7%) and Oct3/4 (12.1%). Conversely, less than 2% of cells were positive for endothelial (CD31, 1.93%) and hematopoietic (CD34, 1.27%) antigens.

### 3.2. Copaiba Oleoresin Promoted the Cell Growth per se and When Combined with Other Biomaterials

**Figure 3:**
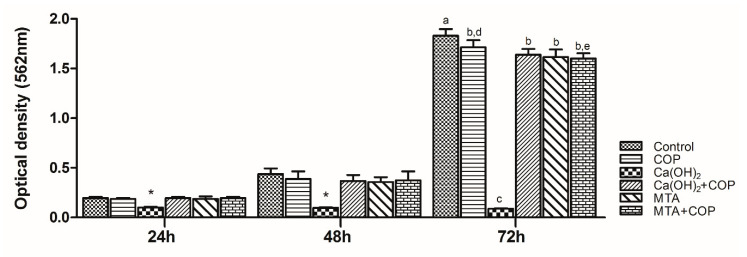
Graphic representation of the mean cell viability (optical density) in all of the experimental groups throughout the experimental time. * The Ca(OH)_2_ group presented the smallest values over the duration of the experiment (*p* < 0.01). Different letters indicate significant differences among the groups at 72 h (*p* < 0.01). Bars indicate the standard error of the mean.

There was significant cell growth in all the groups, except for the Ca(OH)_2_ group. The control group presented the highest viable cell number at 72 h (*p* < 0.01), whereas the Ca(OH)_2_ group presented the lowest number across the whole experimental time (*p* < 0.01). When Ca(OH)_2_ was associated with COP, the proliferative capacity of cells increased significantly (*p* < 0.01) at all time points. There was no difference in the cell viability among the MTA, MTA + COP, and control group. Figure 3 illustrates cell growth in all the experimental groups.

### 3.3. The Association of Copaiba Oleoresin and Mineral Trioxide Aggregate (MTA) Promoted Greater Expression of Genes Associated with Biomineralization Process

**Figure 4:**
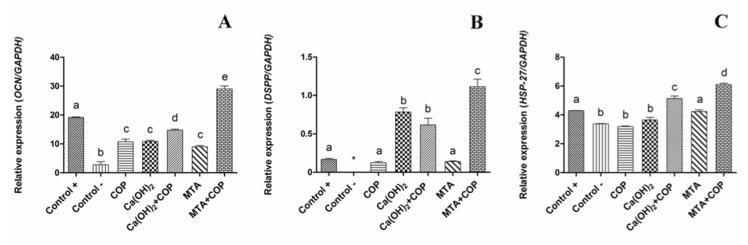
Graphic representation of the relative expression of the *OCN* (**A**), *DSPP* (**B**), and *HSP-27* (**C**) genes in all the experimental groups. Different letters indicate significant differences among the groups for the same gene (*p* < 0.05). Bars indicate the standard error of the mean.

Increased mRNA was detected for the *OCN*, *DSPP*, and *HSP-27* genes in the MTA + COP group, in comparison with all the other groups (*p* < 0.05). *OCN* and *HSP-27* were also upregulated for Ca(OH)_2_ + COP, in comparison with Ca(OH)_2_ solely. Figure 4 shows a graphic representation of the relative expression of the *OCN*, *DSPP*, and *HSP-27* genes.

### 3.4. The Copaiba Improved the Cell Migration When Compared to the Other Biomaterials

**Figure 5:**
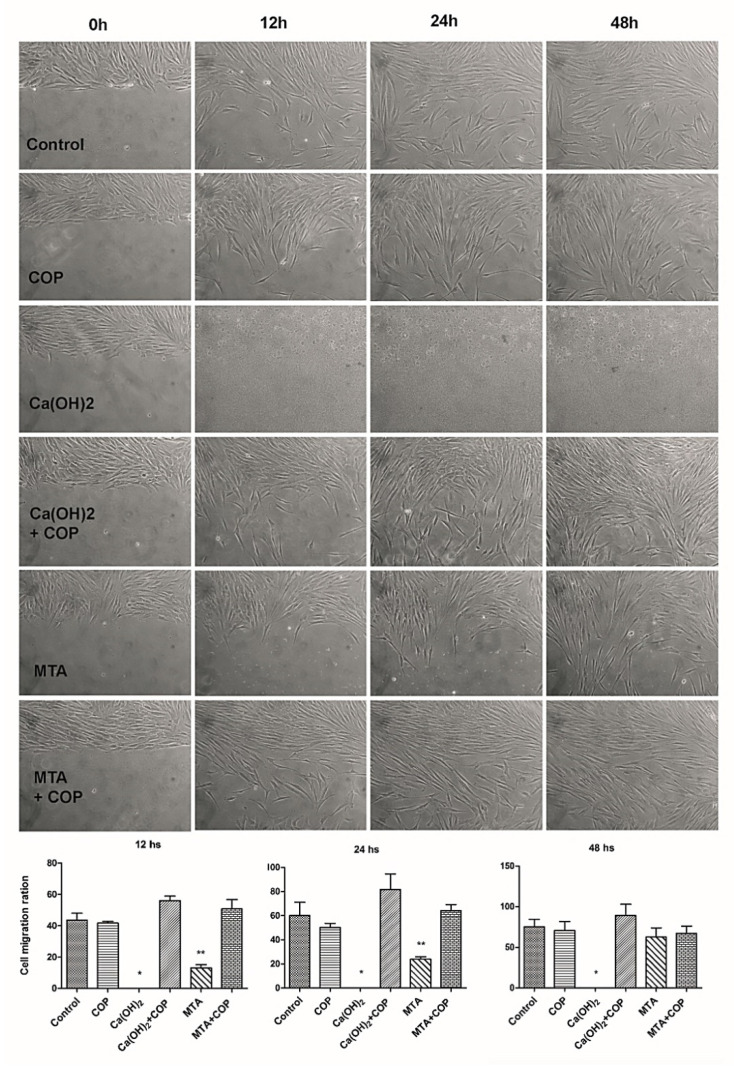
Representative phase microscopy images of cell migration for all the experimental groups over the duration of the experiment (original magnification 100×), along with a graphic representation of the mean cell density in the wound area per time point (cell migration ratio).* No migration was observed; ** significantly smaller than that observed in all the other groups (*p* < 0.05), except for the Ca(OH)2 group at 24 h. Bars indicate the standard error of the mean.

The Ca(OH)_2_ group was the only one that showed no cell migration at any experimental time point. When COP was associated with the other biomaterials [Ca(OH)_2_ and MTA], the migration ratios were significantly higher (*p* < 0.05) at all time points compared with those observed for the biomaterials solely, except for the MTA group, which presented a migration ratio similar to that of the MTA + COP group at 48 h (*p* > 0.05). Figure 5 shows the representative images of cell migration for all the experimental groups over the duration of the experiment, along with a graphic representation of the cell density in the wound area per time point (cell migration ratio).

### 3.5. The Copaiba per se and Combined with Other Biomaterials Promoted the Formation of Mineralization Nodules

**Figure 6:**
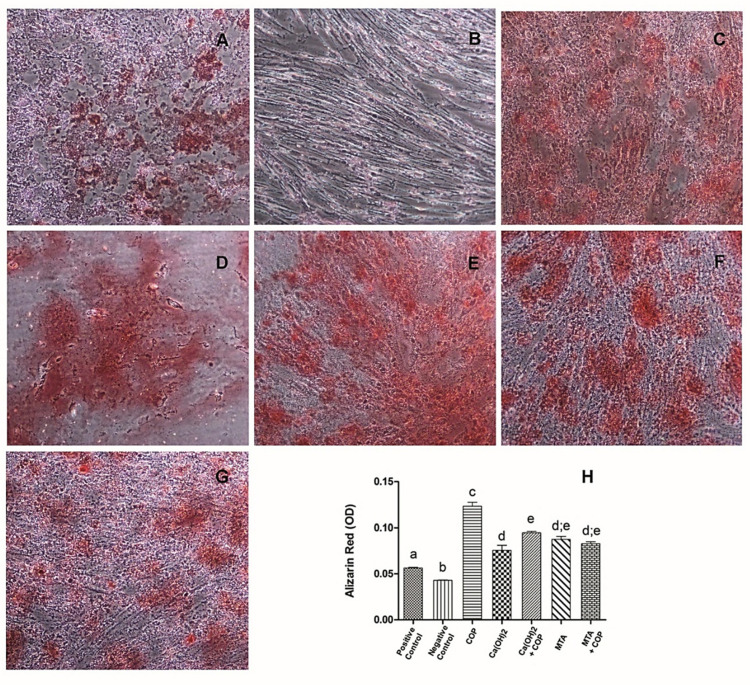
Illustration of cell monolayers submitted to the Alizarin red assay at 21 days after seeding. Representative phase microscopy images of the mineral deposits (in red) in cultured cells of the positive control (**A**); negative control (**B**); Copaiba (COP) (**C**); Ca(OH)_2_ (**D**); Ca(OH)_2_ + COP (**E**); MTA (**F**), and MTA + COP (**G**) (original magnification 100×). Graphic representation of the amount of stain in all the experimental groups (**H**). Different letters indicate significant differences among the groups (*p* < 0.05).

Mineralized nodule formation was observed in all groups. The COP group showed the highest amount of staining (Figure 6C,H; *p* < 0.01), followed by the Ca(OH)_2_ + COP group, whose staining was significantly different from that of the Ca(OH)_2_ group (*p* < 0.01). Figure 6 illustrates the cell monolayers submitted to the Alizarin red assay at 21 days after seeding (Figure 6A–G), along with a graphic representation of the amount of stain in all the experimental groups (Figure 6H). 

## 4. Discussion

The proposition of the present study is that a well-known formulation of the copaiba only or combined with the currently used pulp capping materials [Ca(OH)_2_ and MTA] would improve their biological properties. Our results showed for the first time that copaiba improves the human dental pulp cell response to materials commonly used for direct dental pulp capping in restorative dentistry by increasing the cell proliferation and upregulating the expression of genes related to biomineralization, along with improvements in cell migration and mineralized nodule formation. Our in vitro results demonstrated that copaiba is a natural product with properties capable of optimizing biological properties of products that already exist for pulp capping. Based on these results, one can infer that COP could improve the clinical effectiveness of such dental capping materials.

Dental stem cells are a special type of subpopulation of mesenchymal stem cells (MSCs) that have been shown to possess great potential for multiple biomedical applications, especially for dental tissue regeneration [20]. The discovery of stem cells in the dental pulp of human permanent teeth, termed postnatal dental pulp stem cells (DPSCs) [21], and from the dental pulp of deciduous teeth (stem cells from human exfoliated deciduous teeth (SHEDs)) [22] opened up new horizons to stem cell research. This is mainly because the isolation of these cells is relatively simple and they have the capability to differentiate into cells to replace soft and hard tooth tissue relevant for regenerating the integrity of a damaged tooth [23]. Furthermore, recent studies have shown that SHEDs have even greater proliferative properties than DPSCs and may have a greater disposition for survival than their adult counterparts [24,25]. Thus, both the SHEDs and DPSCs represent adequate models to evaluate in vitro the direct biological effect of biomaterials and/or active molecules in promoting dentin-pulp complex repair mechanisms. The stem cell nature of the cells used in the present study was confirmed by their cell surface expression of MSC-associated markers (CD146, CD105, Nanog, Nestin, Stro-1, and Oct3/4) and lack of expression of endothelial (CD31) and hematopoietic (CD34) antigens.

The literature points out that copaiba has excellent anti-inflammatory pharmacological properties and acts by regulating the excess of pathological inflammation, which leads to tissue preservation and facilitates the regenerative process [14,15,16,17,18]. In addition, the dental pulp capping materials Ca(OH)_2_ and MTA have the ability to create a mineralized tissue barrier. It is important to highlight that the copaiba used in this study and in previous studies from our group [15,16,17,18]) shows phytochemical components with pharmacological properties that justify its therapeutic potential. 

The first results of the present study showed that the substances leached or dissolved from COP and MTA, either individually or in association with each other, exhibited minimal or low cytotoxicity, and did not interfere with cell growth. In fact, other authors have obtained similar results with COP [14,26,27] and with MTA [28,29,30]. In contrast, substances leached from Ca(OH)_2_ inhibited cell growth. This observed cytotoxic effect is still a matter of discussion, in that some authors have confirmed our results [31], whereas others have rejected them because they consider Ca(OH)_2_ to be biocompatible [32]. In fact, the high alkalinity of Ca(OH)_2_ can cause cell death, as demonstrated here; however, in the tissue, this alkalinity is crucial for keeping the exposed area free of microorganisms, which in turn contributes to the formation of a dentin bridge in vivo.

Cell growth inhibition in the Ca(OH)_2_ group is most likely to be related to the irritating nature of this material, which is highly alkaline and soluble in water, and dissociates into calcium and hydroxyl ions, releasing a large quantity of these ions into the conditioned medium. It is known that in vivo, calcium ions react with tissue CO_2_, leading to the formation of calcite granules that can be irritating to the tissue in large amounts [6]. CO_2_ is also present in the atmosphere of the cell culture incubator. In fact, under a phase microscope, deposits of flocculent and birefringent materials were observed at the bottom of the culture plates containing medium conditioned with Ca(OH)_2_. Surprisingly, the association of COP with Ca(OH)_2_ was able to reverse the cytotoxicity of the Ca(OH)_2_, since the cells in this group showed significant growth. One possible explanation for this result would be the greater viscosity of COP, which could have disturbed the ionic dissociation of Ca(OH)_2_. This explanation finds support in the fact that the higher the viscosity of the material, the lower the ionic dissociation of Ca(OH)_2_ [6].

The results of the cytocompatibility assay were in line with those of migration. The only group that did not present migrating cells was the most cytotoxic (i.e., the Ca(OH)_2_ group). The other group that presented a migration ratio smaller than those of the others was the MTA group. These results were probably due to the presence of the calcite granules, which may have hindered the migration process. Corroborating our findings, D’Antò et al. [33] showed a 24-h delay in the migration of bone marrow stem cells under the influence of MTA. Other authors have presented opposite results, showing positive migration of human mesenchymal stem cells in contact with Ca(OH)_2_ [34]. However, these authors used Ca(OH)_2_ in very low concentrations; therefore, the degree of calcite granule formation is likely to be reduced.

The genes studied here were those related to dentin matrix formation and odontoblast differentiation. *OCN* is considered as a marker of mature osteoblasts [34], but is also expressed in dentin. *HSP-27* is closely related to odontoblast differentiation [35]. *DSPP* is highly expressed in dentin, and is also a potential odontogenic differentiation marker [32]. mRNAs of *OCN*, *DSPP*, and *HSP-27* were detected in the MTA + COP group, and the *DSPP* and *HSP-27* genes were expressed in the Ca(OH)_2_ + COP group, indicating that these materials have dentinogenic potential.

The COP group showed the highest amount of mineral deposits. This finding is in line with the research conducted by Lima et al. [27], who pointed out the great potential of COP in forming mineralized tissue during pulp repair in an in vivo study. Other results of the present study corroborated these findings, since a substantial increase in calcium deposit formation was also observed when COP was associated with Ca(OH)_2_, compared with the use of Ca(OH)_2_ alone. These results show that dental pulp capping materials combined with COP are capable of inducing greater functional differentiation of human dental pulp stem cells.

An analysis of the possible factors related to the findings of this study became even more complex when considering that COP presents a range of active pharmacological principles. One possible explanation for this dentinogenic potential is that mineral salts are chemically formed when COP is associated with these biomaterials, considering that its composition includes acid diterpenes (resinous portion), and that Ca(OH)_2_ and MTA are strong bases. In light of the findings by An et al. [36], who pointed out that an increase in calcium ions promotes osteogenic differentiation, the presence of salts formed by combining COP with Ca(OH)_2_ and COP with MTA may be a justification for the upregulation of genes related to dentin matrix formation as well as for odontoblastic differentiation.

The combination of COP with both Ca(OH)_2_ and MTA improved their well-known biostimulation effects, probably due to the viscosity of the COP, which could control the ionic dissociation of such pulp capping materials. Additionally, this viscosity and/or the resinous component of the COP could also make the manipulation of the MTA easier when this material is associated with COP, which will be further explored in future studies. Thus, the biomaterials here proposed could represent an advance toward the search for ideal pulp capping materials with the aim of dental pulp repair. 

Altogether, the data of the present study suggest that the proposed dental material formulations could favor dental tissue repair. However, one has to bear in mind that these results were obtained at the in vitro tests level, which cannot fully represent the actual microenvironment of the tissue repair. Thus, in vivo studies must be done in order to confirm our findings and support a possible future clinical application of these materials.

## 5. Conclusions

The combination of COP with the currently used materials for dental pulp capping [Ca(OH)_2_ and MTA] improved the cell activities related to pulp repair (i.e., cytocompatibility, differentiation, mineralization, and migration) including a protective effect against the cytotoxicity of Ca(OH)_2_. Further investigation should subject these COP combined materials to mechanical tests in vitro as well as to tests of direct pulp capping in vivo.

## 6. Patents

Patent application request (BR 10 2015 015484-4).

## Figures and Tables

**Table 1:** Materials and associations used in this investigation.

Materials	Ca(OH)_2_	White MTA	COP	Ca(OH)_2 +_ COP	White MTA + COP
**Main Components**	Calcium hydroxide	Silicon dioxide, potassium oxide, aluminum oxide, sodium oxide, hematite, sulfur trioxide, calcium oxide, bismuth oxide, magnesium oxide and insoluble residues of crystalline silica, calcium oxide and potassium and sodium sulphates.	δ-elemene; cyclosativene; α-copaene; δ-elemene; cyclosativene; α-copaene; β-elemene; α-gurjunene; β-caryophyllene; trans-α-bergamotene; aromadendrene; epi-β-santalene; α-humulene + (E)-β-farnesene; β-chamigrene; γ-gurjunene; γ-curcumene; β-selinene; α-selinene; (Z)-α-bisabolene; α-bulnesene; β-bisabolene; β-curcumene; β-sesquiphelandrene; (E)-γ-bisabolene; caryophyllene oxide; epi-β-bisabolol and β-bisabolol	Calcium hydroxide, δ-elemene; cyclosativene; α-copaene; δ-elemene; cyclosativene; α-copaene; β-elemene; α-gurjunene; β-caryophyllene; trans-α-bergamotene; aromadendrene; epi-β-santalene; α-humulene + (E)-β-farnesene; β-chamigrene; γ-gurjunene; γ-curcumene; β-selinene; α-selinene; (Z)-α-bisabolene; α-bulnesene; β-bisabolene; β-curcumene; β-sesquiphelandrene; (E)-γ-bisabolene; caryophyllene oxide; epi-β-bisabolol and β-bisabolol	Silicon dioxide, potassium oxide, aluminum oxide, sodium oxide, hematite, sulfur trioxide, calcium oxide, bismuth oxide, magnesium oxide and insoluble residues of crystalline silica, calcium oxide, potassium and sodium sulfates, δ-elemene; cyclosativene; α-copaene; δ-elemene; cyclosativene; α-copaene; β-elemene; α-gurjunene; β-caryophyllene; trans-α-bergamotene; aromadendrene; epi-β-santalene; α-humulene + (E)-β-farnesene; β-chamigrene; γ-gurjunene; γ-curcumene; β-selinene; α-selinene; (Z)-α-bisabolene; α-bulnesene; β-bisabolene; β-curcumene; β-sesquiphelandrene; (E)-γ-bisabolene; caryophyllene oxide; epi-β-bisabolol and β-bisabolol

Composition of copaiba oleoresin (COP) used in the study and characterized by the gas chromatography method; same oil used by Guimarães-Santos et al. (2012) [19] Patent material—INPI BR 10 2013 005551-4, São Paulo, SP, Brazil.
